# Phenolic-glycolipid-1 and lipoarabinomannan preferentially modulate TCR- and CD28-triggered proximal biochemical events, leading to T-cell unresponsiveness in mycobacterial diseases

**DOI:** 10.1186/1476-511X-11-119

**Published:** 2012-09-17

**Authors:** Pradeep Kumar Dagur, Bhawna Sharma, Rajni Upadhyay, Bhavyata Dua, Arshad Rizvi, Naim Akhtar Khan, Vishwa Mohan Katoch, Utpal Sengupta, Beenu Joshi

**Affiliations:** 1 Immunology Laboratory, National JALMA Institute for Leprosy and Other Mycobacterial Diseases (ICMR), Dr.M.Miyazaki Marg, Tajganj, Agra, 282001, India; 2 Molecular & Microbiology Laboratory, National JALMA Institute for Leprosy and Other Mycobacterial Diseases (ICMR), Taj Ganj, 282001, Agra, India; 3 UPRES EA 4183 Lipides & Signalisation Cellulaire, Université de Bourgogne, Faculté des Sciences de la vie, 6, Boulevard Gabriel, Dijon, 21000, France; 4 Present address: Flow-Cytometry Core, NHLBI-NIH, Bethesda, MD, 20892, USA; 5 Present address: Secretary, Department of Health Research and Director-General, ICMR, Ansari Nagar, New Delhi-29, India

**Keywords:** Mycobacteria, PGL-1, Man-LAM, T-cell activation, Anergy

## Abstract

**Background:**

Advanced stages of leprosy show T cell unresponsiveness and lipids of mycobacterial origin are speculated to modulate immune responses in these patients. Present study elucidates the role of phenolicglycolipid (PGL-1) and Mannose-capped lipoarabinomannan (Man-LAM) on TCR- and TCR/CD28- mediated signalling.

**Results:**

We observed that lipid antigens significantly inhibit proximal early signalling events like Zap-70 phosphorylation and calcium mobilization. Interestingly, these antigens preferentially curtailed TCR-triggered early downstream signalling events like p38 phosphorylation whereas potentiated that of Erk1/2. Further, at later stages inhibition of NFAT binding, IL-2 message, CD25 expression and T-cell blastogenesis by PGL-1 and Man-LAM was noted.

**Conclusion:**

Altogether, we report that Man-LAM and PGL-1 preferentially interfere with TCR/CD28-triggered upstream cell signalling events, leading to reduced IL-2 secretion and T-cell blastogenesis which potentially could lead to immunosupression and thus, disease exacerbation, as noted in disease spectrum.

## Background

Mycobacterial infections are leading cause of death worldwide. Tuberculosis, a pulmonary disease, caused by *Mycobacterium tuberculosis (M. tuberculosis),* and leprosy, a neurodegenerative as well as dermal/mucosal disease, caused by *Mycobacerium leprae (M. leprae),* are major mycobacterial diseases. Protective immunity against mycobacterial diseases is mainly due to cell-mediated immunity (CMI) mainly due to T-cells, dendritic cells (DCs), Natural Killer cells (NK cells), macrophages, monocytes, etc. Advance spectrum of mycobacterial infections shows reduced CMI and is widely associated with reduced T-cell responses [1]. These T-cell abnormalities are widely linked with the presence of lipid coat on the mycobacterial cell wall, which account up to 60% of dry weight of mycobacterium [2]. Mannose-capped Lipoarabinomannan (Man-LAM), cell wall-associated highly immunogenic glycolipid, is widely expressed by *M. tuberculosis* and *M. leprae*, whereas Phenolicglycolipid-1, (PGL-1), a polyketide synthase derived phenolicglycolipid, is expressed by *M. leprae*, are among the best-characterized virulence factors of mycobacteria [3].

The lack of protective immunity in patients with advanced stages of mycobacterial infections, leprosy and tuberculosis, could be due to improper microbicidal activity of macrophages, leading to the persistence of viable bacilli within host. As microbicidal activity of macrophages depends on their activation by antigen-specific T-cells, the occurrence of infection could be secondary to interference in the activation process by mycobacteria. Studies have revealed that Man-LAM and PGL-1 not only reduced T-cell proliferation including reduced production of cytokines like IFN-γ, IL-2, IL-4, TNFα, GMCSF, IL-1α, IL-1β, IL-6, IL-8, IL-10 [4-9], but also reduced T-cell activation both *in vitro* as well as *in vivo*[10].

For proper T-cell activation, signalling by TCR and CD28 is required and any alteration in this could lead to T-cell anergy [11]. Though altered T-cell signalling have been reported in tuberculosis [12]), and leprosy [13-15], whereas effect of lipid antigens (Man-LAM and PGL-1) on TCR/CD28- induced signalling, needs a detailed study. Previously, we have demonstrated the response of crude *M. leprae* antigens on the signalling mechanism of T-cells [16] therefore, current study was done to decipher the mechanism of Man-LAM and PGL-1, the lipid components, on signalling events leading to T-cell activation, which still needs documentation.

## Results

The concentration of antigens corresponding to log phase of T-cell proliferation, by lymphocyte transformation assay in PBL and Jurkat T cells (data not shown), was considered as optimal for further signalling experiments. Standardized doses (15 μg/ml for both Man-Lam and PGL-1) were found to have stimulation indices (S.I.) in log phase in patients as well as healthy volunteers ( Additional file 1: Figure S1). The levels of S.I. with antigens (15 μg/ml for both Man-Lam and PGL-1) were significantly lower in patients than healthy volunteers and were significantly lower to the S.I. of PHA and PPD-stimulated cells ( Additional file 1: Figure S2). All the concentrations of antigens were used in some experiments and optimal dose found in LTT was found to be showing maximum effect.

### Man-LAM and PGL-1 preferentially curtail proximal signalling events

**Figure 1:**
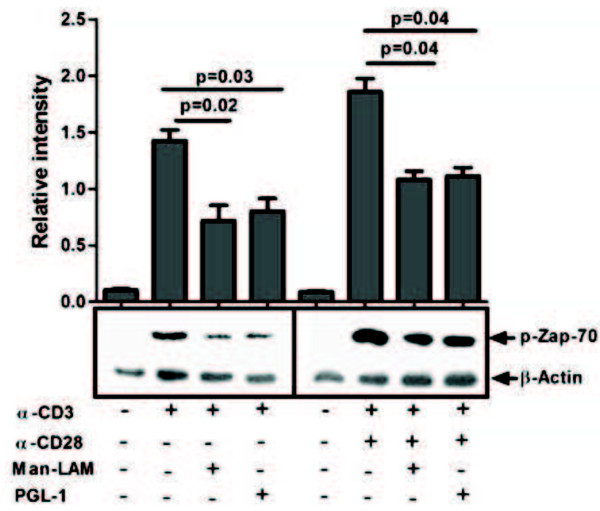
**Man-Lam and PGL-1 inhibit TCR- and TCR/CD28-induced Zap70 phosphorylation.** Untreated or antigen-pretreated Jurkat T-cells were stimulated with α-CD3 or α-CD3 + α-CD28 antibodies, and immunoblots were performed as described in Material and Methods. Membranes were stripped and reprobed with anti-β-actin antibody to confirm equal loading. Histograms represent mean ± SEM values of normalised band intensities of 3 experiments.

**Figure 2:**
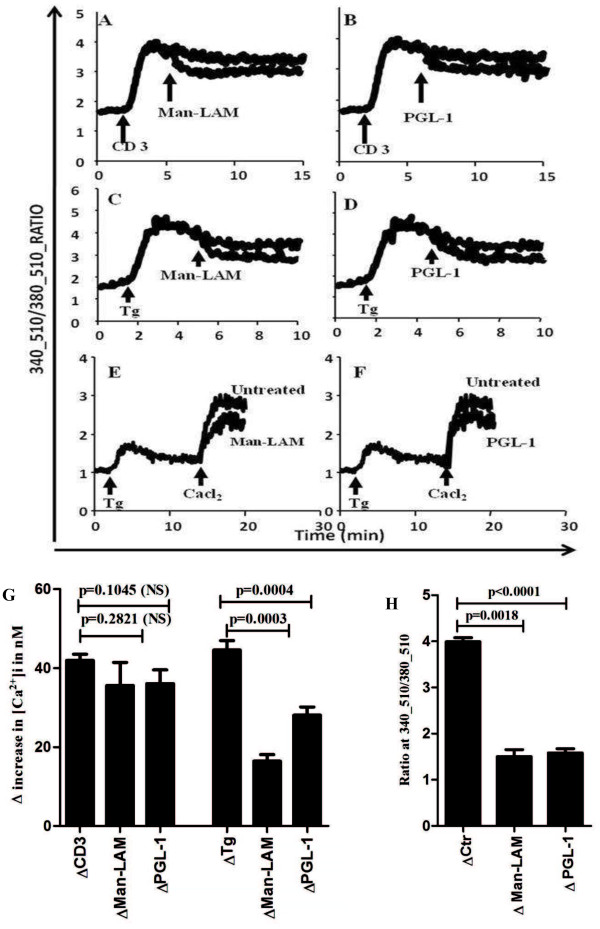
**Mycobacterial lipid antigens diminish increases in [Ca**^**2+**^**]i.** The recordings show 340/380 ratiometric curves, indicating changes in [Ca^2+^]i. The test molecules were added into cuvette during real-time recordings (**A**-**F**). The cells were also pretreated with Man-LAM and PGL-1 and the effect of CD3 (**A** and **B**) and Tg (**C** and **D**) was assessed. The CFCR protocol was used to study the calcium influx, evoked by the addition of TG after Man-LAM (**E**) or PGL-1 (**F**) treatment. Representative curves of three independent experiments are presented here and values derived are shown as mean ± SEM in histograms (**G** and **H**).

TCR trigerred Zap-70 phosphorylation, an early upstream signal, was found to be significantly inhibited by both Man-LAM and PGL-1, which was also evident in presence of costimulation by CD28 (Figure 1). As an increase in free calcium concentration, [Ca^2+^]i, is downstream to Zap-70 activation, we assessed TCR-triggered calcium mobilization by adding Man-LAM and PGL-1 on T-cells after stimulation with anti-CD3 antibody. We noticed that TCR induced calcium mobilization was diminished by two antigens (Figure 2 A, B) though it was not statistically significant (Figure 2G). Thapsigargin (Tg), was used to further elucidate whether these antigens influence the opening of calcium channels as a result of internal store depletion. Significant inhibition by Man-LAM and PGL-1 was noted in refilling of the cytosolic stores from the extracellular environment (Figure 2 C, D and G). Moreover, to directly assess the effects of Man-LAM and PGL-1 on the opening of calcium channels, calcium-free and calcium-reintroduction (CFCR) protocol was designed. For this, first Tg was used to deplete the intercellular calcium stores on EGTA chelated cells and then calcium influx was noted after addition of exogenous calcium. Man-Lam and PGL-1 significantly attenuated exogenous calcium influx, evoked by Tg (Figure 2 E, F and H).

**Figure 3:**
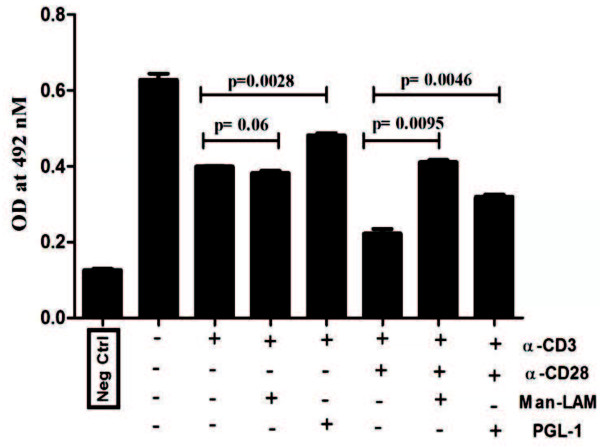
**Man-LAM and PGL-1 modulate PKC activity in the cytosolic fractions of Jurkat T-cells afterα-CD3 orα-CD3+α-CD28 stimulation.** Neg CTRL = Negative control of Kit, Unstim = Unstimulated cells. Values in histogram are mean ± SEM values of OD. Experiments were done in duplicate and were repeated at least three times.

PKC is one of the early markers of T-cell activation and upon activation PKC mobilizes from cytosol to cell membrane. Decreased cytosolic PKC activity was noticed in response to TCR alone or with CD28 stimulation, which indicates signal for cell activation. It was noted that PGL-1 but not Man-LAM increased cytosolic activity as compared to TCR stimulation, whereas, both the antigens increased the PKC activity in cytosolic fractions in TCR/CD28-stimulated cells, which indicates that CD28 mediated PKC activation is preferentially targeted by lipid antigens (Figure 3). Incubation of T-cells alone with either Man-LAM or PGL-1 did not evoke significant changes in phosphorylation levels of Zap-70, cytosolic PKC activity and intracellular calcium levels (data not shown).

### Man-LAM and PGL-1 differentially modulate downstream signalling events and IL-2 gene transcription

**Figure 4:**
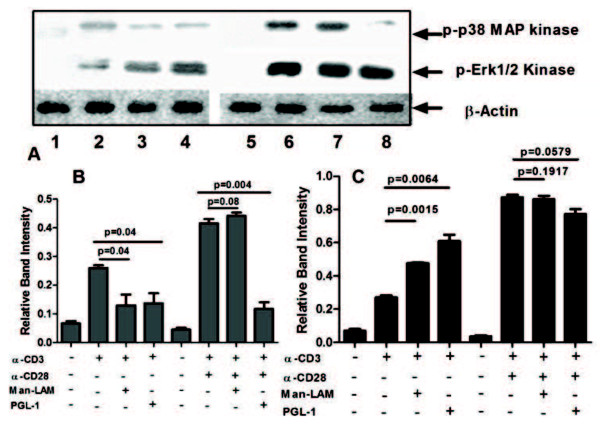
**Man-LAM and PGL-1 inhibit TCR- and TCR/CD28-induced phosphorylation of Erk1/2 and p38MAPkinase.** (**A**): Untreated or antigen pretreated Jurkat T-cells were stimulated with α-CD3 or α-CD3 + α-CD28 antibodies, and immunoblots were performed as described in Material and Methods. Membranes were stripped and reprobed with anti-β-actin antibody to confirm equal loading. Lane 1- Control, 2- α-CD3 stimulated, 3- α-CD3 stimulated in presence of Man-LAM, 4- α-CD3 stimulated in presence of PGL-1, 5- Control, 6- α-CD3 + α-CD28 stimulated, 7- α-CD3 + α-CD28 stimulated in presence of Man-LAM, 8- α-CD3 + α-CD28 stimulated in presence of PGL-1. (**B**): Normalised band intensities of p38MAPkinase phosphorylation (**C**): Normalised band intensities of Erk1/2phosphorylation. Histograms represent mean ± SEM values of band intensities of 3 experiments reproduced at least 3 times independently.

Man-LAM and PGL-1 both significantly inhibited TCR-induced p38MAPK phosphorylation (Figure 4). However, these antigens significantly potentiated Erk1/2 phosphorylation. Similarly, TCR/CD28 trigerred p38MAPK phosphorylation was significantly curtailed by PGL-1 but not by Man-LAM. None of these two antigens significantly modulated TCR/CD28 trigered Erk1/2 phosphorylation (Figure 4 A, B and C).

**Figure 5:**
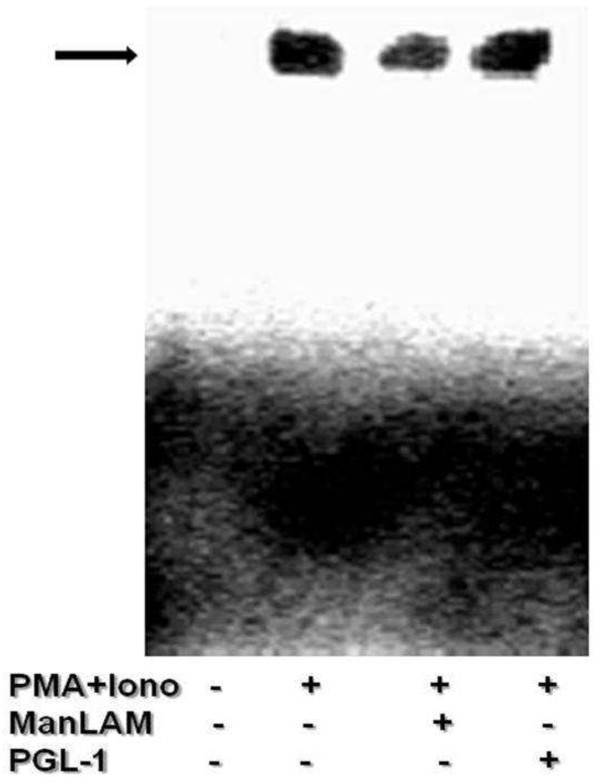
**Man-LAM and PGL-1 modulate DNA binding affinity of NFAT to the promoter of IL-2 cytokine.** Untreated or antigen pretreated Jurkat T-cells were stimulated with PMA/ionomycin and electrophoretic mobility shift assays were performed. Representative blot of experiments were repeated at least three times.

PMA/Ionomycin induces prolonged nuclear translocation of NFAT than TCR/CD28 stimulation; therefore DNA binding of NFAT on IL-2 promoter was assessed on PMA/ionomycin stimulation of antigen pretreated cells. We observed that Man-LAM but not PGL-1 pretreated cells showed decreased DNA binding activity of NFAT (Figure 5).

**Figure 6:**
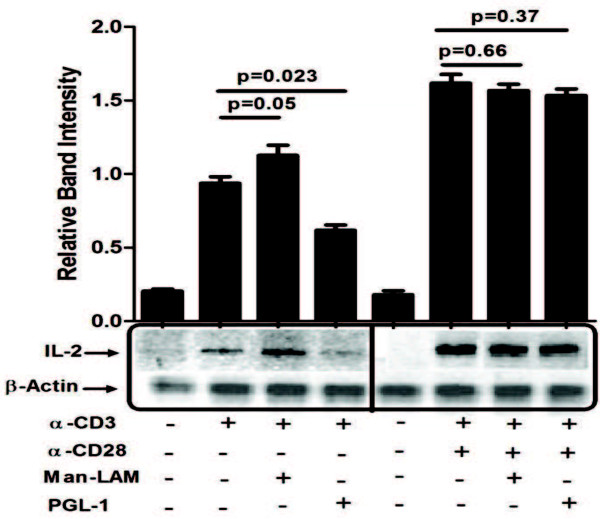
**Man-Lam and PGL-1 inhibit expression of IL-2 mRNA.** Untreated or antigen pretreated Jurkat T-cells were stimulated with α-CD3 or α-CD3 + α-CD28 antibodies, and RT-PCR were performed as described. Equal amplification of β-actin was performed as control.

As all of these signalling events are linked to IL-2 gene transcription, its transcription was studied further. We observed that PGL-1 significantly inhibited; however, Man-LAM potentiated TCR-stimualted IL-2 gene transcription. No significant effect on TCR/CD28 stimulated IL-2 mRNA levels was noted by both antigens (Figure 6).

### Both Man-LAM and PGL-1 downregulate CD25 expression

**Figure 7:**
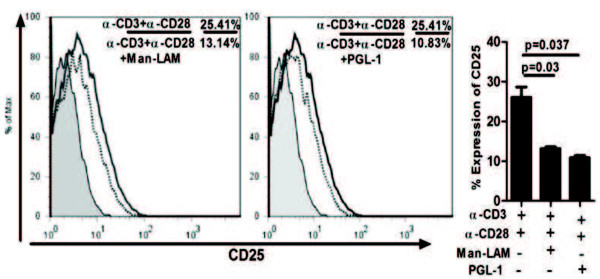
**Mycobacterial lipid antigens inhibit TCR/CD28 stimulated IL-2Rα (CD25 expression.** Flowcytometric analysis showing histogram overlay of CD25 receptor of untreated or antigen pretreated Jurkat T-cells stimulated with α-CD3 + α-CD28 antibodies. Histograms are representative plots of experiments repeated at least 3 times independently. Here solid bold curve represent expression on α-CD3 + α-CD28 stimulation while dotted bold lines represent expression on stimulation with α-CD3 + α-CD28 plus antigens and shaded histogram shows expression by unstimulated cells. Bar diagrams represent mean ± SEM values of percent positive cells expressing CD25.

Induction of CD25 (IL-2Ra) by CD3 + CD28 stimulation (25.4%) was significantly inhibited by Man-LAM (13.14%) and PGL-1 (10.83%) as compared to stimulation of CD3 + CD28 (Figure 7).

### Man-LAM and PGL-1 inhibits production of IL-2 cytokine and T-cell blastogenesis in healthy individuals

**Figure 8:**
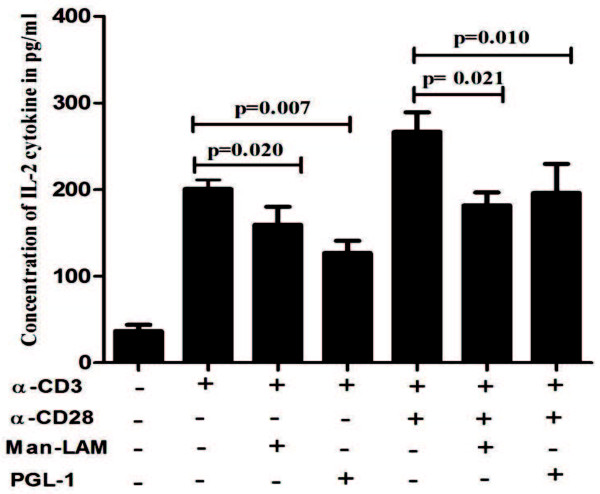
**Man-LAM and PGL-1 inhibit TCR- and TCR/CD28-induced secretion of IL-2 cytokine in PBMCs of healthy individuals.** Bar diagram showing mean ± SEM concentration of IL-2 in pg/ml in the culture supernatant of PBMCs of healthy individuals (N = 5) after stimulation with α-CD3 or α-CD3 + α-CD28 in presence or absence of Man-LAM and PGL-1 at the end of 48 h. Experiments were performed in triplicates.

**Figure 9:**
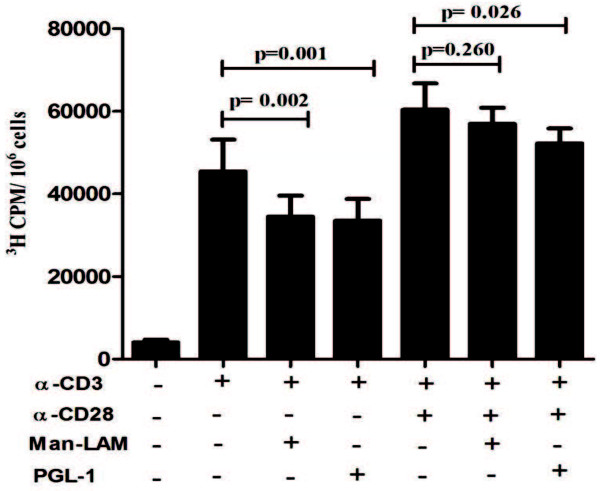
**Man-LAM and PGL-1 inhibit TCR- and TCR/CD28-induced T-cell proliferation.** Bar diagram showing mean ± SEM of tritiated thymidine (3 H) uptake by PBMCs of healthy individuals (N = 5) after stimulation with α-CD3 or α-CD3 + α-CD28 in presence or absence of Man-LAM and PGL-1 at the end of 72 hrs. Experiments were performed in triplicates.

Both Man-LAM and PGL-1 inhibited not only TCR-trigerred but also TCR/CD28-induced IL-2 production significantly by PBL derived T-cells of healthy individuals (Figure 8). Furthermore, TCR-induced T-cell blastogenesis was also inhibited significantly by these two antigens, whereas CD28 costimulation could overcome this inhibition (Figure 9).

## Discussion

Proper T-cell activation is a prerequisite for effective cell mediated immune (CMI) response against intracellular pathogens like mycobateria, leishmania, etc. Advanced stages of mycobacterial infections like tuberculosis and leprosy show loss of T-cell functions, including reduced IL-2 production, which could be due to improper T-cell activation [17]. IL-2 production is necessary for T-cell activation, which is induced by TCR and CD28 receptors signalling [18]. As lipids are known to differentially modulate T-cell signalling [19], and mycobacterial species have high contents of lipid in their cell wall [3], this study was designed to study the impact of mycobacterial lipid antigens, (Man-LAM and PGL-1) on T-cell activation and hence the mechanistic role of lipids in modulation of T-cell physiology in mycobacterial diseases which still needs to be delineated.

We observed that both Man-LAM and PGL-1 significantly inhibited either TCR alone or TCR/CD28- trigerred Zap-70 phosphorylation. Mahon *et al.*, 2009 [20] also confirms this finding where they used whole lipid fraction of *M. tuberculosis* and found reduced Zap-70 phosphorylation. This reveals defective proximal TCR mediated signalling by mycobacterial lipid fractions Man-LAM and PGL-1.

Further, downstream second messenger such as calcium influx was investigated. Calcium signalling is central to most of the cellular activities including antigen presentation, cellular motility and cell activation. For T-cells, calcium signalling is directly linked with the outcome of immune response, like turnover and secretion of cytokines, cell proliferation, etc. [21]. Our observation shows that though Man-LAM and PGL-1 did not significantly curtail TCR-trigerred calcium influx, but they curtailed refilling of intracellular calcium stores significantly. Furthermore, it was observed that these lipid antigens also curtailed overall influx of calcium by inhibiting opening of calcium channels as evidenced by antigen- mediated inhibition in ionomycin triggered influx and CFCR protocols. These observations strongly suggest that mycobacterial lipid antigens diminish the opening of plasma membrane associated calcium channels, which are the result of [Ca^+2^i depletion from internal stores. These findings are in concordance with our previous results where we have reported that soluble fractions of *M. leprae* lysate blocked opening of store operated CRAC channels [22]. Present study establishes that mycobaterial lipid fractions alters calcium signalling and consequently plays a major role in the pathogenesis. Indeed, it is known that *M. tuberculosis*[23] and *M. leprae*[16] curtails intracellular calcium levels which could be linked with reduced phagosome-lysosome fusion, thus for increased survival of *M. tuberculosis* in macrophages [24]. Interestingly, PBMCs from patients with mycobacterial infection have been reported to have reduced cytosolic [Ca^+2^i concentrations [13,15,25] which could be due to inhibition in calcium mobilization across plasma membrane in lymphocytes by mycobacterial lipid antigens, as elucidated by series of calcium mobilization experiments performed in the present study.

PKC translocates from cytosolic fraction to membrane fraction upon activation by upstream signals such as Zap-70/PLCγ [26], therefore, we analysed PKC activity in T cells. Significantly higher cytosolic PKC levels in lipid antigen-treated T-cells was observed, indicating the inhibitory effect of mycobacterial lipids on PKC trnslocation which could be due to inhibition of upstream signals. Altogether these findings allude that Man-LAM and PGL-1 play a crucial role in inhibition of proximal TCR signalling.

We further studied downstream signalling events viz activation of MAPkinases and nuclear translocation of transcription factor NFAT, important events for both cytokine production and cell activation [27]. Interestingly we found that both Man-LAM and PGL-1 curtailed TCR-triggered p38Mapkinase phosphorylation but potentiated Erk1/2 phosphorylation, whereas on TCR/CD28 stimulation, this trend could be reversed in Man-LAM but not in PGL-1 treated p38Mapk phosphorylation, inhibitory effect of Man-LAM could be nullified after co-stimulation through CD28. This is in contrast to findings with whole *M. leprae* lysate where we have shown inhibition of Erk1/2 and p38 both [16]. Our findings of differential modulation of MAPkinases by mycobacterial lipid antigens are supported by Tapinos and Rambukanna [28], where they have shown that live leprosy bacilli hijack cellular signalling machinery by potentiating Erk1/2 phosphorylation but not p38 in Schwann cells, which further promotes cell proliferation which could aid in spreading of bacilli. Therefore, it could be inferred that lipid antigens reduce cellular toxicity of mycobacterial lysates, as reported elsewhere [29], which is in contrast to inhibitory effect exerted by mycobacterial whole cell soluble lysate on Erk1/2 phosphorylation in T-cells [22].

NFAT binds to sites in the regulatory regions of several cytokine genes including IL-2. Upon TCR stimulation dephosphorylated form of NFAT translocates to nucleus [26,27]. We observed inhibition by Man-LAM but not by PGL-1 on DNA binding of NFAT. Defects in NFAT activity has also been found in T regulatory cells [30]. Also Garg *et al.*[31] reported that Man-LAM led to expansion of T regulatory cells. These reports further strengthens that lipid antigens could drive hyporesponsive state of T-cells by suppressing the induction and propagation of TCR-initiated signals to control IL-2 production and cell proliferation as reported here.

Our results indicate that Man-LAM and PGL-1 significantly alter TCR/CD28- triggered signalling, leading to diminished activity of Zap-70, calcium influx and PKC mobilization, differential phosphorylation of MAPKs and DNA binding of NFAT. As all these events lead to production of IL-2 cytokine, therefore, production of IL-2 at mRNA level was studied. It was noted that while PGL-1 inhibited expression of TCR triggered IL-2 mRNA, inhibition induced by Man-LAM was not significantly different. This effect was recovered with CD28 costimulation and TCR stimulation, further indicating that CD28 signalling was not primarily targeted by Man-LAM and PGL-1. Expression of CD25 (IL-2Rα) is necessary for the binding of IL-2 and thus helps in self maintenance by T-cells to avoid anergy [32]. Therefore, its expression was evaluated after activation through CD3 + CD28 ligation in presence or absence of Man-LAM and PGL-1. Interestingly, these antigens curtailed TCR/CD28 mediated CD25 expression significantly. Whole cell lysates of *M. tuberculosis*[33] and *M. leprae*[16] have been shown to attenuate CD25 expression, which could be specifically due to lipid fractions present in them as reported in this study. Upregulation of CD25 is involved in the autocrine proliferative response [32], whereas downregulation of CD25 could also be additive in imposing anergic state in T-cells. Above results indicate that Man-LAM and PGL-1 could lead hyporesponsive state of T-cells by suppressing the transduction of TCR/CD28-induced signals for IL-2 production and hence T-cell proliferation.

To substantiate the hypothesis generated by using Jurkat T-cells and to further assess the effect of mycobacterial lipid antigens T-cell proliferation and IL-2 cytokine production in healthy subjects, we performed the bioassays using PBMCs derived from healthy individuals. Man-LAM and PGL-1 were found to significantly inhibit TCR mediated IL-2 secretion and T cell blastogenesis both. However, co-stimulation by CD28 could help cells to proliferate (survival) but not for IL-2 production, which further suggests that TCR triggered signalling is primarily targeted by Man-LAM and PGL-1, over CD28- induced signalling.

## Conclusion

Altogether our findings establish that Man-LAM preferentially inhibit TCR-mediated proximal signaling events like Zap70 phosphorylation, calcium influx, PKC activation, but potentiate phosphorylation of Erk1/2 over p38mapk, whereas overall major effect on various signalling pathways was contributed by PGL-1 as both TCR and CD28 co-stimulatory pathways are involved. It is possible that the ratio of PGL-1 and Man-LAM decides the modulation of T cell responses in leprosy leading to a state of hyporesponsiveness by upregulating Erk1/2 phosphorylation over p38mapk. Further, there is reduction of IL-2 production at protein levels and also its uptake by inhibiting CD25 expression in T cells which eventually leads to reduced T-cell proliferation. These events do not show hypervirulence nature of mycobacteria as evident by Sinsimer *et al.*, 2008. Altogether, these events favour longer intracellular survival for the bacilli by diminishing T-cell coordinated CMI, an indispensable arm of immunity to clear intracellular infection, leading to disease progression, as noticed in advance stages of mycobacterial infections such as tuberculosis and leprosy.

## Materials and methods

Jurkat T-cell line was procured from national cell repository, India and peripheral blood lymphocytes (PBL) were isolated from buffy coats from healthy donors and leprosy patients (both tuberculoid and lepromatous) by density gradient separation. Cells were maintained in C-RPMI (RPMI-1640 + 10%FBS + 2 mM-L-Glutamine + 1X antibiotic-antimycotic cocktail) at 37°C and 5%CO_2_ in a humidified incubator. Study was approved by institutional ethical committee and subjects were included after getting their informed consent.

### Chemicals and antigens

General reagents were purchased from Sigma. Fura-2-AM was from Calbiochem, USA. Anti-human CD3 (HIT3-α), anti-human CD28 (CD28.2), anti-human CD25-PECy5, were from Becton Dickinson,USA. P-p38MAPK and P-Erk1/2 antibodies were from Cell Signalling Technology, USA. β-actin and peroxidase-labelled secondary antibodies were from Santacruz Technologies, USA. Tritiated-thymidine was from BARC, India whereas ECL reagent from Millipore, USA. PGL-1 was procured from Dr. Brennan, under Leprosy research support (NIH-N01-A1-25469), whereas Man-LAM from Dr. Spencer, under TB Vaccine Testing and Research Material contract (NIH, NIAID NOI-AI-40091), Colorado State University, USA.

### Quantification of transmembrane Ca^2+^ mobilisation

Jurkat T-cells (2x10^6^/ml) were incubated with Fura-2/AM dye at 1 μM for 30 min at 37°C in loading buffer. (pH 7.4 (in mM): CaCl_2_, 1.2; NaCl, 110 ; NaHCO_3_, 25; KCl, 5.4; KH_2_PO_4_, 0.4; HEPES, 20; MgCl_2_, 0.8;Na_2_HPO_4_, 0.33). Loaded cells were washed three times (2000 g x 10 min) and resuspended in loading buffer. [Ca^2+^i was measured by adding the test molecules in cuvette without interruption in reading as reported elsewhere [16,34].

### Treatment and activation of cells

Serum starved Jurkat T-cells (5x10^6^/ml) were incubated or not with Man-LAM or PGL-1 for two hours, then stimulated or not with anti-CD3 antibody (10 μg/ml) and/or anti-CD28 antibody (5 μg/ml) at 4°C, followed by cross linking using GAM-IgG (5 μg/ml) at 37°C as per Kim and White [35].

### Analysis of PKC activation

After 15 min of stimulation at 37°C Jurkat T-cells were lysed with 50 μl of buffer (EDTA, 1 mM; EGTA, 1 mM; HEPES, 20 mM pH 7.3; NaCl, 0.15 mM; Triton X-100, 1%; glycerol, 10%; PMSF, 1 mM; Na_3_VO_4_, 2 mM; anti-protease cocktail). After centrifugation (13,000 x g for 1 min), clear cell lysates were used immediately or stored at −80°C. Cytosolic protein contents were estimated with Bradford reagent. PKC activity in the cytosolic fractions was measured in duplicate using protein kinase assay kit as per manufacturer’s instructions (Calbiochem, USA).

### Western blotting of Zap-70 and MAPKs

Denatured proteins (30 μg) were separated by SDS-PAGE (10%) and transferred on polyvinylidine difluoride (PVDF) membranes. Detection of phosphorylated forms of Zap-70, p38 MAPK and Erk1/2 was done using 2 μg/ml antibody in TBS-BSA (2.5%) with overnight incubation at 4°C. After washing with TBST (TBS + 0.05% Tween-20), membranes were incubated with secondary antibodies for 30 min at RT and peroxidase activity was detected on X-ray sheets using ECL reagents. Membranes were stripped and further reprobed for β-Actin to confirm equal loading. Quantity One^TM^ software (BioRAD, USA) was used for densitometric analysis of protein bands.

### Electrophoretic mobility shift assay (EMSA)

Nuclear extract was prepared (as per Schrieber et al.) [36], from serum starved Jurkat T-cells preincubated with Man-LAM and PGL-1 for 2 h and then stimulated with PMA (5nM) and ionomycin (2 μM) for 2 h. The nuclear extract was either used fresh or was frozen in aliquots at -70°C till further use. The sequence of the oligonucleotides used for NFAT was 5'-GATCTTTACATTGGAAAATTTTAT-3' [37] and was biotinylated using 3’-end DNA labelling kit (Pierce Endogen, USA) and annealed for 2 h at RT. Binding reactions were carried out using 20 fmol of biotin-end-labelled target DNA with 4 μg of nuclear extract for 20 min at RT as per kit’s instructions [LightShift^TM^ chemiluminiscent EMSA kit, Pierce Endogen, USA]. Assays were run on a pre run native 4% PAGE gel in 0.5x Tris borate/EDTA at 100 V before transferring to a charged nylon membrane (Millipore, USA). Band shifts were detected after crosslinking (120 mJ/cm^2^) the transferred DNAs to the membrane as per kit instructions.

### Transcription of IL-2 gene

Similarly pretreated Jurkat T-cells were stimulated with anti-CD3 ± anti-CD28 and cross-linked as described above. Total RNA was isolated using Trizol-R (Invitrogen, USA). 0.5 μg of total RNA was reverse-transcribed using RT-PCR kit (Bangalore Genei, Bengaluru, India) and PCR amplification for IL-2 and β-actin was performed as described elsewhere (Dagur et al., 2010). Amplicons were electrophoresed on 1.5% agarose gel containing ethidium bromide. Gel images were captured and band densities were quantified using Gel Documentation System (Bio Rad, USA).

### Expression of CD25 by flow cytometry

T-cells pre-incubated or not with antigens were stimulated with TCR/CD28 for 18 h at 37°C in humidified 5% CO_2_ incubator. Expression of CD25 was measured by using α-humanCD25Cy5PE antibodies. Samples were acquired on FACSAria^TM^ flow cytometer equipped with FACSDIVA^TM^ software (BD Biosciences). FlowJo software was used to analyse the acquired data (Treestar, USA).

### Effect of lipid antigens on IL-2 cytokine production and T-cell proliferation

PBMCs were isolated from healthy volunteers and were incubated with α-CD3 antibody alone or with α-CD28 antibody at 4°C for 15 min. Then cells were given a quick wash and were added on GAM-IgG (5ug/ml) pre-coated wells (2x10^5^ per well in C-RPMI) supplemented or not with Man-LAM and PGL-1. At the end of 48 h IL-2 was estimated in supernatant by ELISA as per manufacturer's instructions (Bender Medsystems, Austria). Whereas tritiated thymidine (1 μCi/well) was added in the culture for last 16 h and radioactivity was read in liquid scintillation counter (LKB Wallac, Netherlands) at the end of 72 h of incubation.

### Statistical analysis

Data were analysed using Graphpad Prism-3.02 software (San Diego, CA, USA) and Mean ± SEM were calculated. Paired student’s t test was used to compare values with in a group and P value < 0.05 was considered as significant.

## Abbreviations

IL-2: Interleukin 2; PGL-1: Phenolicglycolipid of *M. leprae*; Man-LAM: Mannose capped Lipoarabinomannan of *M. leprae*; [Ca^+2^]i: Free intracellular calcium concentrations.

## Competing interests

The authors declare that they have no competing interests.

## Authors’ contributions

PKD performed all the experiments. BS and BD helped in LTT and western blots. RU and AR helped in LTT and dose standardization. NAK, VMK and USG helped in concept and writing of the manuscript. BJ conceived and executed the study. All authors read and approved the final manuscript.

## Supplementary Material

Additional file 1 **Figure S1.** Dose optimisation curve of Man-LAM and PGL-1 by using PBMCs of healthy individuals by MTT dye uptake assay (Different concentration of 2.5, 5, 7.5, 10, 15, 20, 25 μg/ml of antigens were taken). Dose corresponding to log phase was taken as optimal dose for further assays. **Figure S2.** Lymphoproliferative responses of healthy and leprosy patients (TT/BT and BL/LL) using H3-thymidine uptake assay. Bar diagram showing mean ± SEM of stimulation indices (S.I) of Tuberculoid (TT/BT) (N = 10), Lepromatous (BL/LL) patients (N = 5) and healthy individuals (N = 10) after stimulation of their PBMC’s with optimized doses of PHA, PPD, Man-LAM and PGL-1. S.I was calculated according to the formula: $S.I.=\frac{Mean\;counts\;per\;minute\;of\;experimental\;wells}{Mean\;counts\;per\;minute\;of\;control\;wells}$ Click here for file
