# Supplementary figures and images for: Phenolic-glycolipid-1 and lipoarabinomannan preferentially modulate TCR- and CD28-triggered proximal biochemical events, leading to T-cell unresponsiveness in mycobacterial diseases
Source: Lipids Health Dis. 2012 Sep 17;11:119. doi: 10.1186/1476-511X-11-119 (PMC3477116; doi:10.1186/1476-511X-11-119)

Figure:-S1

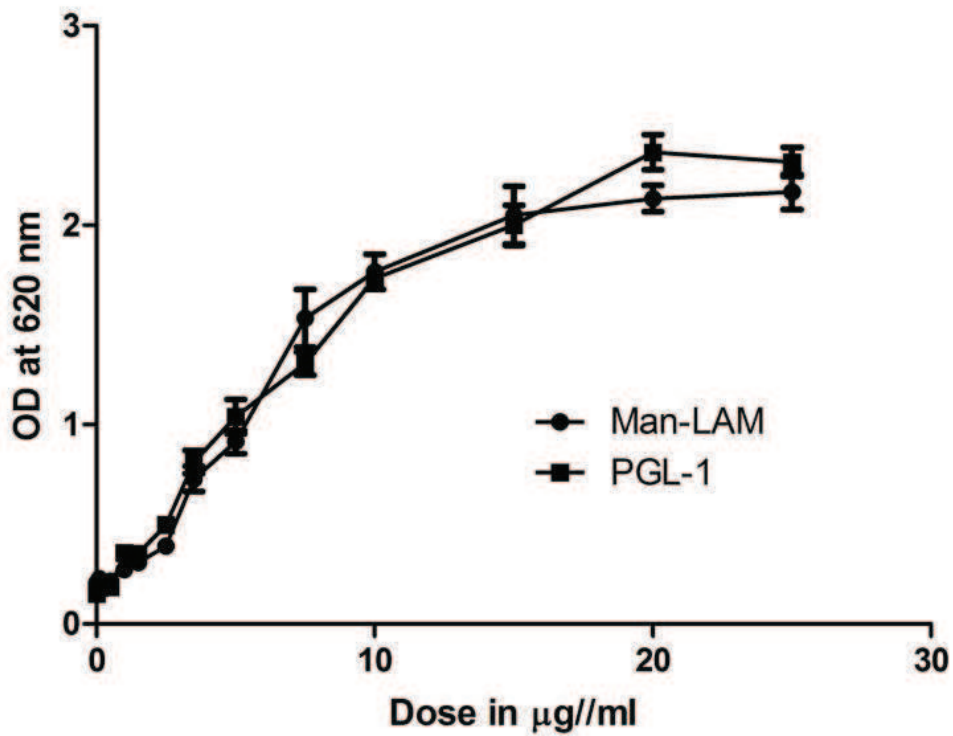

Figure:-S2

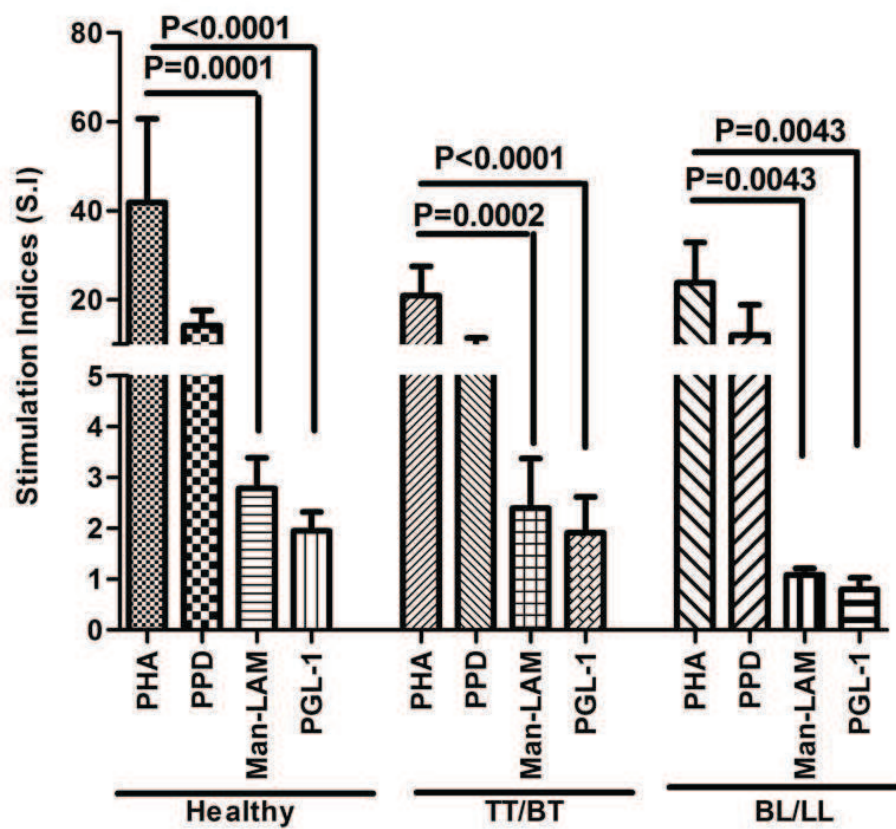

Supplement: Additional file 1 — Figure S1. Dose optimisation curve of Man-LAM and PGL-1 by using PBMCs of healthy individuals by MTT dye uptake assay (Different concentration of 2.5, 5, 7.5, 10, 15, 20, 25 μg/ml of antigens were taken). Dose corresponding to log phase was taken as optimal dose for further assays. Figure S2. Lymphoproliferative responses of healthy and leprosy patients (TT/BT and BL/LL) using H3-thymidine uptake assay. Bar diagram showing mean ± SEM of stimulation indices (S.I) of Tuberculoid (TT/BT) (N = 10), Lepromatous (BL/LL) patients (N = 5) and healthy individuals (N = 10) after stimulation of their PBMC’s with optimized doses of PHA, PPD, Man-LAM and PGL-1. S.I was calculated according to the formula: S.I.=MeancountsperminuteofexperimentalwellsMeancountsperminuteofcontrolwells [file 1476-511X-11-119-S1.pdf]
